# Evaluation of an Accelerated Workflow for Surveillance of ESBL (CTX-M)-Producing *Escherichia coli* Using Amplicon-Based Next-Generation Sequencing and Automated Analysis

**DOI:** 10.3390/microorganisms6010006

**Published:** 2018-01-11

**Authors:** Nilay Peker, John W. A. Rossen, Ruud H. Deurenberg, Paula C. Langereis, Erwin G. C. Raangs, Jan A. Kluytmans, Alexander W. Friedrich, Jacobien Veenemans, Bhanu Sinha

**Affiliations:** 1 Department of Medical Microbiology, University Medical Center Groningen, University of Groningen, 9713 GZ Groningen, The Netherlands; n.peker@umcg.nl (N.P.); ruuddeurenberg@home.nl (R.H.D.); p.c.langereis@gmail.com (P.C.L.); g.c.raangs@umcg.nl (E.G.C.R.); alex.friedrich@umcg.nl (A.W.F.); b.sinha@umcg.nl (B.S.); 2 Laboratory for Microbiology and Infection Control, Amphia Hospital, 4818 CK Breda, The Netherlands; jankluytmans@gmail.com (J.A.K.); jacobien.veenemans@gmail.com (J.V.)

**Keywords:** amplicon-based next-generation sequencing, *E. coli*, beta-lactamases, ESBL, CTX-M, outbreak surveillance, molecular diagnostics

## Abstract

Outbreak management of extended spectrum *β*-lactamase (ESBL)-producing pathogens requires rapid and accurate diagnosis. However, conventional screening is slow and labor-intensive. The vast majority of the screened samples are negative and detection of non-outbreak-related resistant micro-organisms often complicates outbreak management. In a CTX-M-15-producing *Escherichia coli* outbreak, 149 fecal samples and rectal eSwabs were collected by a cross-sectional survey in a Dutch nursing home. Samples were processed by routine diagnostic methods. Retrospectively, ESBL-producing bacteria and resistance genes were detected directly from eSwab medium by an accelerated workflow without prior enrichment cultures by an amplicon-based next-generation sequencing (NGS) method, and culture. A total of 27 (18.1%) samples were positive in either test. Sensitivity for CTX-M detection was 96.3% for the phenotypic method and 85.2% for the NGS method, and the specificity was 100% for both methods, as confirmed by micro-array. This resulted in a positive predictive value (PPV) of 100% for both methods, and a negative predictive value (NPV) of 99.2% and 96.8% for the phenotypic method and the NGS method, respectively. Time to result was four days and 14 h for the phenotypic method and the NGS method, respectively. In conclusion, the sensitivity without enrichment shows promising results for further use of amplicon-based NGS for screening during outbreaks.

## 1. Introduction

The introduction of cephalosporins as a treatment option for infections caused by *Enterobacteriaceae* was followed by the emergence of extended spectrum β-lactamases (ESBL)-producing pathogens. ESBLs are enzymes that can hydrolyze third generation cephalosporins produced by several *Enterobacteriaceae*, including *Klebsiella pneumoniae* and *Escherichia coli* [1,2,3]. Among the different classes of ESBLs (TEM, SHV, CTX-M among others), the prevalence of CTX-M carrying *Enterobacteriaceae* is rising [4,5,6,7,8,9,10].

Outbreaks with ESBL-producing *Enterobacteriaceae* are an increasing problem [11]. Outbreak management, in general, requires rapid diagnosis for optimum quality of infection control measures. Rapid action in order to limit the consequences and to contain the dissemination of ESBL-producing *Enterobacteriaceae* is of utmost importance. However, conventional screening is usually slow and labor-intensive, and therefore does not meet the requirements for a rapid diagnostic test. The combination of efficient logistics, an optimal workflow and accelerated diagnostic procedures has the potential to maximally support infection control during management and prevention of outbreaks. One important option for acceleration is the use of molecular diagnostics on direct patient material, since it circumvents the primary requirement for bacterial growth. With such methods, relevant (partial) results can be obtained earlier. This has two positive aspects, provided that the sensitivity, and subsequently, the negative predictive value (NPV) is high. One aspect is that patients with a negative result can be rapidly cleared, and the other aspect is that labor intensive culture-based work can be focused on the relatively small proportion of positive samples. An additional advantage is that molecular diagnostics can be automated more easily with already commercially available equipment.

However, microbiological diagnostics for surveillance faces at least two additional challenges which largely depend on the regional epidemiology of the studied pathogens. In a low prevalence setting for a given micro-organism, most samples will be negative, thus leading to a large number of negative tests which still bind laboratory capacity and resources. By contrast, in a high prevalence setting, there will be substantial numbers of ‘by-catch’, i.e., accidental detection of micro-organisms which are unrelated to the outbreak. To safely exclude negative patients from further screening, it is desirable to have a rapid and easy screening test with a high sensitivity. In addition, a test specific for the outbreak strain to avoid background noise in the screening will be extremely helpful in guiding infection prevention measures [11]. Additionally, feces samples, often used in diagnostics, are an extremely complex and bacteria-rich matrix.

In this study, an amplicon-based next-generation sequencing method, commercialized as the Pathogenica Hospital Acquired Infection (HAI) BioDetection System, was used for detection and typing of pathogens and resistance genes [12,13]. The HAI BioDetection system involves sequencing of short DNA regions of interest which are targeted by a set of probes to bind specific genes [12,13]. However, the original protocol includes an enrichment step, and in a standard workflow without 24/7 dedicated staff, the advantage of rapid detection is largely lost with this enrichment step. Therefore, in the present study, the amplicon-based next-generation sequencing method was used for the rapid exclusion of negative samples and the detection of positive samples, without the pre-enrichment step, directly from primary samples.

## 2. Materials and Methods 

### 2.1. Samples

During a CTX-M-15-producing *E. coli* outbreak in a long-term care facility in the south of The Netherlands, fecal samples or rectal eSwabs (Copan Diagnostics, Brescia, Italy) were collected from all residents (*n* = 149) during a cross-sectional survey in October 2013. 

### 2.2. Phenotypic Detection and Confirmation by Micro-Array 

Samples were tested for the presence of ESBL-producing bacteria using a selective tryptic soy broth containing 0.25 mg/L cefotaxime and 8 mg/L vancomycin that was sub-cultured on a selective McConkey medium divided in two sections containing 400 mg/L cloxacillin, 64 mg/L vancomycin, plus on either of the two sections 1 mg/L ceftazidime or 1 mg/L cefotaxime, respectively (ESBL Screening Agar ([EbSA]), AlphaOmega, The Hague, The Netherlands). All media were incubated overnight at 35 to 37 °C. Species identification and susceptibility testing were performed on all isolates that grew on either section of the EbSA medium using matrix assisted laser desorption/ionisation—time of flight mass spectrometry (MALDI-TOF MS) with the Biotyper software package (Bruker, Karlsruhe, Germany) and VITEK 2 (bioMérieux, Marcy l’ Etoile, France), respectively. CTX-M β-lactamases have higher hydrolyzing activities against cefotaxime than ceftazidime which is usually very well hydrolyzed by SHV type ESBLs [14]. However, several CTX-M-1 group enzymes also hydrolyze ceftazidime and cefepime efficiently [15]. Thus, the presence of ESBLs was confirmed phenotypically with the combination disk diffusion test (CDT) for cefotaxime, ceftazidime and/or cefepime, either alone or in combination with clavulanic acid (Rosco, Taastrup, Denmark). Isolates were considered ESBL positive when the inhibition zone around the disk was 5 mm or larger for the disk containing the antibiotic in combination with clavulanic acid, as compared to the disk without clavulanic acid. For every non-duplicate ESBL-positive isolate of each patient, a Check-MDR CT103 micro-array (Checkpoints, Wageningen, The Netherlands) was performed to confirm the presence of ESBL resistance genes.

### 2.3. Next-Generation Sequencing 

Different DNA extraction methods, including the DNeasy Blood and Tissue kit (Qiagen, Gaithersburg, MD, USA), QIAamp DNA Stool Mini kit (Qiagen, Hilden, Germany), automatic DNA isolation using the NucliSENS easyMag (bioMérieux, Marcy l’Etoile, France) and UltraClean Microbial DNA Isolation kit (MoBio, Carlsbad, CA, USA), were evaluated on six feces samples and six rectal eSwabs. DNA concentration and purity were assessed using a NanoDrop 2000 c spectrophotometer (Thermo Fisher Scientific, Waltham, MA, USA) and the Qubit double-stranded DNA (dsDNA) HS and BR assay kits (Life Technologies, Carlsbad, CA, USA). Since the UltraClean Microbial DNA Isolation kit appeared to be the most efficient method for this purpose, it was used for the DNA isolation of the collected samples. 

The feces and rectal eSwabs were thawed from −80 °C at room temperature. For DNA isolation, 500 μL eSwab medium was taken from the swab and centrifuged in a 1.5 mL Eppendorf tube for 1 min at 14,000× *g*. The supernatant was used for DNA isolation, using the UltraClean^®^ Microbial DNA Isolation Kit according to the procedure as described by the manufacturer.

Library preparation was performed as described in the procedure of the HAI BioDetection Kit (BioInnovation Solutions SA, Lausanne, Switzerland), which utilizes targeted set of around 300 probes to bind and amplify the specific DNA regions, but omitting the enrichment step of 12 to 16 h pre-incubation. To optimize the DNA concentration for the clonal amplification, the library concentration was diluted in two steps and measured using the Qubit 2.0 Fluorometer (Life Technologies, Blijswijk, The Netherlands). In the first dilution step, the library was diluted to 1 ng/μL in water, and in the second dilution step, the library was diluted 1:528. Template preparation was carried out using the Ion PGM Template OT2 200 Kit (Life Technologies, Carlsbad, CA, USA) and the PGM Ion Torrent (Life Technologies, Carlsbad, CA, USA) was used for sequencing of the library. Subsequently, automatic data analysis was performed using the HAI software version 1.2 (BioInnovation Solutions SA, Lausanne, Switzerland) which is based on a mapping approach. The software compares sequencing data of the each sample to both the Genbank database and to Pathogenica’s constantly updated sequencing database to determine the best matches to microbial strains and resistance genes as indicated in the manufacturer’s user guide (Pathogenica HAI BioDetection Kit Software Version 1.2.0 User Guide 2012). The HAI BioDetection kit can detect 12 bacteria (*Staphylococcus aureus*, coagulase-negative *Staphylococci*, *Enterococcus faecalis*, *Enterococcus faecium*, *Acinetobacter baumannii*, *Enterobacter cloacae*, *Enterobacter aerogenes*, *Klebsiella pneumoniae*, *Escherichia coli*, *Proteus mirabilis*, *Pseudomonas aeruginosa*, and *Clostridium difficile*), as well as 18 antibiotic resistance genes (CARB, CMY, CTX-M, GES, IMP, KPC, NDM, ampC, OXA, PER, SHV, VEB, VIM, ermA, vanA, vanB, mecA and mexA). However, the kit cannot differentiate between wild-type (WT) and ESBL alleles for SHV.

### 2.4. Statistical Methods 

The non-parametric test for related samples of binary data was performed using the software package SPSS version 20.0 (IBM Corporation, New York, NY, USA).

## 3. Results

### 3.1. DNA Isolation 

Pilot experiments were performed prior to the study in order to optimize the workflow and the use of the software for data analyses. During these initial experiments, it appeared that the method used for DNA isolation was crucial for the performance of the amplicon-based next-generation sequencing method. Due to the low microbial DNA concentrations in the rectal swabs, we were not able to determine the quality of the isolated DNA (A260/A280 ratios). Therefore, sequencing results, i.e., the number of positive samples and the number of reads, were used to determine which isolation kit could be best used for our study. The QIAamp DNA Stool Mini Kit did not yield any results. Automated DNA isolation using the NucliSENS easyMag yielded results for five of 12 samples. The DNeasy Blood and Tissue kit gave results for eight of 12 samples, whereas the UltraClean^®^ Microbial DNA Isolation kit gave results for all samples with higher numbers of reads compared to the DNeasy Blood and Tissue kit (Supplementary Table S1). 

### 3.2. Detection of CTX-M-15-ESBL-Producing E. coli 

The conventional phenotypic method (culture + CDT) detected an ESBL-producing *E. coli* isolate in 26 of 149 (17.4%) of the samples. Subsequent microarray analysis revealed these were all carrying the CTX-M-15 gene. The amplicon-based next-generation sequencing method on direct samples detected an *E. coli* and a CTX-M gene in 23 of 149 (15.4%) samples, including one sample that was tested negative with the phenotypic method. In total, 27 of 149 (18.1%) patients were positive for *E. coli* and a CTX-M gene with at least one method (Table 1).

### 3.3. Statistical Analyses 

A pooled sensitivity was assumed as the gold standard. Samples that tested positive in either test were confirmed as CTX-M-15 ESBL-positive by micro-array, and hence 100% specificity was inferred for both tests. Sensitivity for the CTX-M detection was 26 of 27 (96.3%) for the phenotypic method and 23 of 27 (85.2%) for the amplicon-based next-generation sequencing method. The specificity of 100% resulted in a positive predictive value (PPV) of 100% for both methods. The NPVs were 99.2% and 96.8% for the phenotypic method and the amplicon-based next-generation sequencing method, respectively (Table 2). The proportion of samples tested positive was not statistically different (*p* = 0.375) between the two methods (17.4% versus 15.4%), resulting in an absolute difference of 2 percentage points.

### 3.4. Discrepant Results 

Five samples showed discrepant results depending on the method used. In one sample, a CTX-M gene was detected by the amplicon-based next-generation sequencing method only, but no resistance was detected by the phenotypic method. In contrast, four samples tested CTX-M positive with the phenotypic method and subsequent micro-array, were negative using the amplicon-based next-generation sequencing method. Therefore, the positive CTX-M samples missed with the amplicon-based next-generation sequencing method were tested again in three ways: (1) samples were enriched in Brain heart infusion (BHI) medium for 16 h before being plated on blood agar plates and being tested by the amplicon-based next-generation sequencing method, (2) a fresh DNA isolation was performed from the samples followed by testing using the amplicon-based next-generation sequencing method, and (3) the previously isolated DNA was again used for the amplicon-based next-generation sequencing method. In addition, all isolates were identified by MALDI-TOF and phenotypic antibiotic susceptibility testing was performed to confirm the presence of ESBL-producing *E. coli*. Only the first approach revealed one extra sample positive for *E. coli* and CTX-M. The second and third method did not result in any additional positive samples.

### 3.5. Times to Result

**Figure 1:**
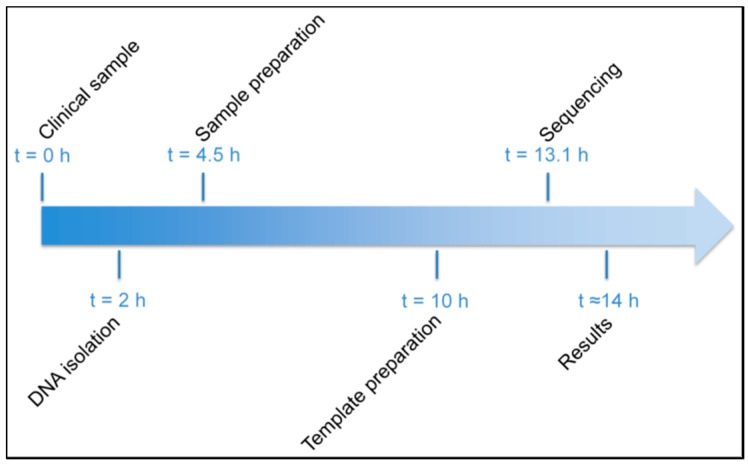
Accelerated workflow for the amplicon-based next-generation sequencing method used in the present study.

The times to result for the phenotypic culture-based method and the amplicon-based next-generation sequencing method were four days and 14 h, respectively, if no more than 24 samples were processed in parallel using the amplicon-based next-generation sequencing method (Figure 1).

## 4. Discussion

In the present study, we used an amplicon-based next-generation sequencing method for the rapid detection of negative samples and the detection of expected positive patients using an accelerated workflow without enrichment culture, a step included in the original protocol of the manufacturer. This study showed that the sensitivity for CTX-M detection with the phenotypic method was 96.3%, and with the amplicon-based next-generation sequencing method 85.2%. The latter is promising, but still lower than desirable and a reported sensitivity of 98% for the same kit when used directly on DNA extracted from isolates [12]. The specificity was assumed 100% for both methods, resulting in a PPV of 100% for both methods and a NPV of 99.2% and 96.8% for the phenotypic method and the amplicon-based next-generation sequencing method, respectively. The proportion of samples tested positive, 17.4% versus 15.4%, was not statistically different between the two methods. 

From the initial experiments, the extraction protocol used to isolate the DNA from the samples appeared to be crucial for the subsequent performance of the amplicon-based next-generation sequencing method. The difference in the results using different DNA extraction methods could be due to a difference in the quality of the DNA obtained by the specific kits. Unfortunately, DNA concentrations in our e-swab samples were too low to measure the quality of the DNA. 

The sensitivity with the accelerated workflow with no enrichment showed promising results. However, one sample was found to be positive for *E. coli* and CTX-M using the amplicon-based next-generation sequencing method, and this was not observed by the phenotypic method. Furthermore, four samples from which a CTX-M-producing *E. coli* could be detected using the phenotypic method were negative using the amplicon-based next-generation sequencing method. Such discrepant results have not been observed in a previous study, in which the results of the amplicon-based next-generation sequencing method were compared with results obtained by PCR and sequencing [13]. However, another study also observed the failure of the detection of CTX-M in one isolate using the amplicon-based next-generation sequencing method [12]. These discrepancies could be explained by the loss of the plasmid carrying the CTX-M gene along with performing these two methods retrospectively from thawed samples. Indeed, the microarray assay was not able to detect the CTX-M gene strongly suggesting that the plasmid carrying the gene is not present (anymore). Although the positive CTX-M samples missed by the amplicon-based next-generation sequencing method were tested again by enriching on an unselective BHI media, only one extra positive sample was detected. It is already known that, enrichment of resistant bacteria in an unselective medium may result in isolates losing their resistance genes, located on plasmids over time. Further optimization of the DNA isolation from direct material (such as eSwabs) by reducing human DNA, as well as optimal sample handling, may further increase the sensitivity of the amplicon-based next-generation sequencing method. An automated DNA isolation method is preferred to standardize the workflow and to reduce hands-on time. Furthermore, the amplicon-based next-generation sequencing method can be used for sequence-based typing of isolates in addition to identification of species and resistance genes [13]. Also, this targeted-based sequencing approach is more efficient and cost effective for the processing of high number of samples in comparison to conventional PCR [13]. As well as, generation of smaller data sets makes the bioinformatics analysis easier and shorter in processing time compared to a whole genome sequencing approach. 

Although the automated data analysis of the provided software has several advantages, as, e.g., its simplicity and generating an easy-to-read output report, it also limits in depth analyses of the sequence data as users are not able to adjust any parameters. This may affect quality aspects of the data analysis. However, the raw sequencing reads can be downloaded and processed with any other bioinformatics tool. 

Despite the somewhat lower sensitivity of the amplicon-based next-generation sequencing method compared to the phenotypic method, the time to result for the amplicon-based next-generation sequencing method was only 15% of that of the phenotypic method (14 h and four days, respectively). However, the trade-off between sensitivity and speed of having results with either the phenotypic method or the amplicon-based next-generation sequencing method is inevitable. It is still likely to exclude ESBL-positive samples by having an ideal target of a NPV of 100% whereas no false positives were obtained either one of the methods in this study. In conclusion, methods such as the amplicon-based next-generation sequencing method should eventually find their way to the microbiology diagnostic laboratories. However, further studies are required to shorten duration of the library preparation and the run on the PGM Ion Torrent instrument. We conclude that a molecular screening method for outbreak management offers several advantages, most prominently, a substantial increase in the time to result.

## Figures and Tables

**Table 1:** Detection of CTX-M-15 ESBL genes by the amplicon-based next-generation sequencing method (NGS) on direct patient material, compared to conventional method (culture + CDT, followed by microarray).

	Phenotypic/Microarray Method		Total
	No. (%) Positive	No. (%) Negative	
NGS positive	22 (84.6)	1 (0.8)	23 (100)
NGS negative	4 (15.4)	122 (99.2)	126 (100)
Total	26 (100)	123 (100)	149 (100)

**Table 2:** Sensitivity, specificity, PPV and NPV for the amplicon-based next-generation sequencing method (NGS) and the phenotypic/microarray method.

	Sensitivity (% [No.])	Specificity (% [No.])	PPV (% [No.])	NPV (% [No.])
NGS	85.2 (23)	100 (122)	100 (23/23)	96.8 (122/126)
Phenotypic/microarray	96.3 (26)	100 (122)	100 (26/26)	99.2 (122/123)
Total	100 (27)	100 (122)	-	-

## Supplementary Materials

The following are available online at www.mdpi.com/2076-2607/6/1/6/s1, Table S1: Comparison of different DNA isolation methods.

Supplementary File 1
