# Supplementary material for: Evaluation of an Accelerated Workflow for Surveillance of ESBL (CTX-M)-Producing Escherichia coli Using Amplicon-Based Next-Generation Sequencing and Automated Analysis
Source: Microorganisms. 2018 Jan 11;6(1):6. doi: 10.3390/microorganisms6010006 (PMC5874620; doi:10.3390/microorganisms6010006)
Supplement: Supplementary File 1 [file microorganisms-06-00006-s001.pdf]

Table S1. Comparison of different DNA isolation methods.

Comparison 1

Isolation Method	Total # of samples	# of samples tested postive	Total # of reads
MoBio	12	12	2756250
bioMerieux	12	5	2359974

Comparison 2

Isolation Method	Total # of samples	# of samples tested postive	Total # of reads
MoBio	12	12	1840428
Qiagen	12	8	262843
